# Prognostic value of pre-therapeutic FDG-PET radiomic analysis in gastro-esophageal junction cancer

**DOI:** 10.1038/s41598-023-31587-8

**Published:** 2023-04-08

**Authors:** Karim Amrane, Philippe Thuillier, David Bourhis, Coline Le Meur, Chloe Quere, Jean-Christophe Leclere, Marc Ferec, Veronique Jestin-Le Tallec, Laurent Doucet, Pierre Alemany, Pierre-Yves Salaun, Jean-Philippe Metges, Ulrike Schick, Ronan Abgral

**Affiliations:** 1 Department of Oncology, Regional Hospital of Morlaix, Morlaix, France; 2 Department of Endocrinology, University Hospital of Brest, Brest, France; 3 UMR Inserm 1304 GETBO, IFR 148, University of Western Brittany, Brest, France; 4 Department of Nuclear Medicine, University Hospital of Brest, 2 Avenue Foch, 29609 Brest Cedex, France; 5 Department of Oncology, University Hospital of Brest, Brest, France; 6 Department of Otorhinolaryngology, University Hospital of Brest, Brest, France; 7 Department of Gastroenterology, Regional Hospital of Morlaix, Morlaix, France; 8 Department of Oncology, Pasteur Clinic, Brest, France; 9 Department of Pathology, University Hospital of Brest, Brest, France; 10 Department of Pathology, Ouestpathology Brest, Brest, France; 11 Department of Radiotherapy, University Hospital of Brest, Brest, France

**Keywords:** Biomarkers, Oncology

## Abstract

The main aim of this study was to evaluate the prognostic value of radiomic approach in pre-therapeutic ^18^F-fluorodeoxyglucose positron-emission tomography (FDG-PET/CT) in a large cohort of patients with gastro-esophageal junction cancer (GEJC). This was a retrospective monocenter study including 97 consecutive patients with GEJC who underwent a pre-therapeutic FDG-PET and were followed up for 3 years. Standard first-order radiomic PET indices including SUV_max_, SUV_mean_, SUV_peak_, MTV and TLG and 32 textural features (TFs) were calculated using LIFEx software on PET imaging. Prognostic significance of these parameters was assessed in univariate and multivariate analysis. Relapse-free survival (RFS) and overall survival (OS) were respectively chosen as primary and secondary endpoints. An internal validation cohort was used by randomly drawing one-third of included patients. The main characteristics of this cohort were: median age of 65 years [41–88], sex ratio H/F = 83/14, 81.5% of patients with a histopathology of adenocarcinoma and 43.3% with a stage IV disease. The median follow-up was 28.5 months [4.2–108.5]. Seventy-seven (79.4%) patients had locoregional or distant progression or recurrence and 71 (73.2%) died. In univariate analysis, SUV_mean_, Histogram-Entropy and 2 TFs (GLCM-Homogeneity and GLCM-Energy) were significantly correlated with RFS and OS, as well as 2 others TFs (GLRLM-LRE and GLRLM-GLNU) with OS only. In multivariate analysis, Histogram-Entropy remained an independent prognostic factor of both RFS and OS whereas SUV_mean_ was an independent prognostic factor of OS only. These results were partially confirmed in our internal validation cohort of 33 patients. Our results suggest that radiomic approach reveals independent prognostic factors for survival in patients with GEJC.

## Introduction

Gastro-esophageal junction cancers (GEJC) develop near the anatomical junction between esophagus and stomach. Their incidence has recently increased in several countries with 456.000 new cases worldwide annually. These tumors result from 2 distinct etiologies: severe atrophic gastritis (diffuse subtype resulting in adenocarcinoma (ADC)) and Barrett’s esophagus (intestinal subtype resulting in squamous cell carcinoma (SCC)). Their prognosis is very poor despite aggressive treatment strategies including surgery and chemo-radiotherapy (CRT). Indeed, in a recent European review of 111.006 GEJC, overall survival (OS) was 13.9% at 5 years^1^ and 75% of all recurrences occur in the first year after surgery^2^. Common prognostic factors have been highlighted in the literature: age, sex, nutritional status, performance status (PS), tumor size, lymph node involvement, distant metastasis, anatomical subsite (Siewert classification), surgery of the primary lesion, time to progression after first-line therapy, histology (SCC versus ADC) and human epidermal growth factor receptor-2 (HER2) status^3^. Nevertheless, despite careful evaluation of these clinical factors, it remains difficult to reliably predict patient’s outcome after a selected treatment.

Pre-therapeutic ^18^F-fluorodeoxyglucose positron-emission tomography (FDG-PET/CT) is currently indicated for staging of locally advanced GEJC^4^. Furthermore, several studies have shown that tumoral uptake assessed by different standard quantitative static (SUV = standardized absorption value) and volumetric (MTV = metabolic tumor volume, TLG = total lesion glycolysis) parameters was a prognostic factor of survival in various solid cancers, including esophageal and gastric locations^5–7^. Nevertheless, no real defined threshold is validated in routine practice to select patients with poor prognosis.

Recently, radiomic approach was developed to characterize tumor heterogeneity by extracting textural features (TFs) from different imaging modalities including PET. SUV, MTV and TLG may be considered by definition as first-order radiomic features but do not provide informations concerning the spatial distribution of voxel values. Thus, second and higher order parameters allow modeling this spatial relationship with numerous TFs^8^. This image-based approach could lead to more personalized therapy in this particular cancer entity with poor prognosis. Indeed, it appears necessary to better classify patients at baseline into different predictive subgroups of treatment outcomes to guide the decision of potential intensification with a higher toxicity risk. The textural analysis of tumor in pre-therapeutic FDG-PET/CT may so be helpful. To date, only few studies have assessed the prognostic value of pre-therapeutic FDG-PET/CT radiomic analysis in esophageal and gastric cancers^9,10^. In a cohort of 403 patients with esophageal cancer including 110 GEJC, Foley and al. found that only first-order parameters such as Histogram-Energy (p = 0.011) and Histogram-Kurtosis (p = 0.017) were independent prognostic factors of OS, unlike textural features^11^. To our knowledge, no study focusing specifically on GEJC was ever conducted.

The aim of this study was to assess the prognostic value of pre-therapeutic FDG-PET/CT radiomic analysis, including TFs, in a large cohort of patients with GEJC.

## Materials and methods

### Patients

Consecutive patients with a histologically proven GEJC who underwent a pre-treatment FDG-PET/CT were retrospectively included from June 2012 to June 2018. Patients with previous GEJ malignancy were excluded. The study was conducted in accordance with the Declaration of Helsinki and was approved by the French Advisory Committee on Information Processing in Health Research (CCTIRS). If patients were alive, they received a written information on the study to express their non-opposition; for deceased patients, an exemption of information was obtained from CCTIRS.

### Data acquisition

FDG-PET/CT images were acquired on two Biograph mCT PET/CT 64 scanner system (Siemens Healthineers^©^, Erlangen, Germany) with the same technical setting. Patients fasted 6 h before intravenous injection of approximately 3 MBq/kg of FDG. Following injection, patients remained in a quiet room for approximately 60 min before acquisition.

At first, CT scan was obtained in the craniocaudal direction using a whole-body protocol, just after injection of intravenous iodine contrast agent (1.5 mL/kg), unless contraindicated. The CT consisted in a 64-slice multidetector-row spiral scanner with the following standard parameters: transverse field of view of 700 mm, collimation of 16 × 1.2 mm, pitch = 1, tube voltage of 120 kV, and effective tube current of 80 mAs.

Then, the PET images were acquired in the craniocaudal direction using a whole-body protocol (2 min per step) and were reconstructed using an ordered subset expectation maximization (OSEM) algorithm (True X = point spread function + time of flight compensation ordered subset expectation maximization-3D). Images were corrected for random coincidences, scatter and attenuation using the CT scan data and were smoothed with a Gaussian filter (full-width at half-maximum = 2 mm). The reconstruction transaxial matrix size was 200 × 200 voxels and the voxel size was 4.07 × 4.07 × 2 mm.

### Images analysis

All primary tumors were analyzed using LIFEx software (www.lifexsoft.org)^12^ on PET imaging. Each primary lesion was segmented by a single nuclear medicine physician using a fixed SUV threshold method consisting in delineating a 3D contour around voxels equal to or greater than 40% of SUV_max_. In this volume of interest (VOI), intensities of FDG uptake were resampled with an absolute method of 64 discrete values and bounds set to 0 and 30 SUV values, corresponding to the typical range of tumor SUVs encountered in GEJC^6,7^. Forty-seven radiomic features including 7 conventional (SUV_max_, SUV_mean_, SUV_peak_, SUV_min_, SUV_std_, MTV and TLG), 8 histogram and shape-based parameters (first-order PET indices) and 32 textural features = TFs (second and higher-order indices) were recorded as detailed in supplemental data. TFs could only be computed by the software for VOI equal to or greater than 64 voxels and containing only 1 cluster. For VOI containing more than 1 cluster, the most representative one was manually selected by the operator based on its uptake intensity and volume.

### Follow-up

Clinical data including age, sex, histological type (SCC or ADC), human epidermal growth factor receptor-2 (HER2) expression, metastatic status and initial pathologic stage according to the American Joint Committee on Cancer classification (AJCC) were recorded. Patients were treated in accordance with standard guidelines after a gastro-intestinal multidisciplinary board and were clinically followed up for at least 1 year to evaluate the relapse free survival (RFS) and overall survival (OS). As recommended by National Comprehensive Cancer Network, European Society for Medical Oncology (ESMO) and French National Society of Gastroenterology, clinical follow-up consisted in a standard digestive examination, including inspection and palpation and an examination of internal structures by a mirror and a flexible endoscope every 2 months during the first year and 3 months during the second year after treatment.

RFS, defined as the duration from diagnosis to disease progression or death of any cause, or to date of last follow-up, was chosen as primary endpoint. OS which was defined as the duration from diagnosis to death or date of last follow-up, was chosen as secondary endpoint.

### Statistics

Descriptive statistics were used to characterize the cohort. Quantitative variables were described as median and range. Qualitative variables were presented as number (n) and percentage (%).

A preliminary correlation analysis between first-order radiomic PET indices and TFs was estimated using a Pearson test for quantitative variables and a Chi-square test for qualitative variables. Groups of highly correlated PET parameters (r > 0.8) were extracted with the same method as suggested by Orlhac et al.^13^. One parameter from each independent group was selected for analysis only if area under the curve AUC was statistically correlated with survival.

An univariate analysis was first performed to test significance of the following factors: age > vs ≤ 65 years^3^, sex (M vs. F), histology (ADC vs. SCC), AJCC stage (I-II vs. III-IV), treatment (surgery vs. no surgery) and selected radiomic features. Receiver-operating characteristic (ROC) curve analysis was performed to determine the optimal cut-off value that divided all patients into 2 subgroups of poor or good prognosis for each selected factor. Area under the curve (AUC), sensitivity (Se), specificity (Sp) and accuracy (Acc) were reported. The Kaplan Meier method was used to estimate survival probabilities. A log-rank test was used to compare the RFS and OS distribution.

A multivariate analysis using Cox proportional hazard model was then performed to assess the potential independent effect of prognostic factors outlined in univariate analysis.

An additional subgroup analysis with HER2 status (overexpression versus no expression) in adenocarcinoma histological subtypes was performed to test parameters highlighted in the multivariate analysis.

An internal validation cohort was used by randomly drawing one-third of included patients.

The significance level of p-value was 0.05. All statistics were realized with XLSTAT 2020 life software (Addinsoft, Paris, France).

## Results

### Population

Between June 2012 and June 2018, 110 patients with GEJC who underwent a pre-therapeutic FDG-PET/CT were identified and then followed-up until September 2021. In 13 cases, an undersized segmented tumor VOI could not allow textural indices calculation by the software. Injection of an intravenous iodinated contrast agent was performed in 64 of the 97 patients analyzed. Both first-order radiomic PET indices and TFs were calculated in 97 patients (male = 83, female = 14) with a median age of 65 years (range, 41 to 88). The main characteristics of the cohort are described in Table 1. Seventy-nine patients (81.5%) presented an ADC histology, among whom 31.2% had an HER2 overexpression status and 32.9% were Siewert type II at inclusion.

**Table 1:** Characteristics of patients at baseline.

Characteristics	No of patients (n = 97)
Age years, median (range)	65 (41–88)
Sex (male/female)	83/14
Histology, n (%)	
Squamous cell carcinoma	18 (18.5)
Adenocarcinoma	79 (81.5)
AJCC stage, n (%)	
I (T1a N0 M0)/(T1b N0 M0)	07 (7.2)
II (T2 N0 M0)/(T1 N1 M0)/(T3 N0 M0)	17 (17.5)
III (T1 N2 M0)/(T2 N1 M0)/(T2 N2 M0)/(T3 N1/N2 M0)/(T4a N0/N1 M0)	31 (32.0)
IV (T4a N2 M0)/(T4b Nx M0)/(Tx N3 M0)/(Tx, Nx, M1)	42 (43.3)
Treatment, n (%)	
Surgery alone	10 (10.3)
Neo and adjuvant treatment + surgery	39 (40.2)
Radiochemotherapy (RCT)	18 (18.5)
Chemotherapy alone	28 (28.9)
Best supportive care	02 (2.1)
Lost to follow-up	16 (16.5)
	Adenocarcinoma (n = 79)
Siewert classification (adenocarcinoma type), n (%)	
I	34 (43.0)
II	26 (32.9)
III	19 (24.1)
Mutations (for adenocarcinoma), n (%)	
HER2 +	25 (31.6)
HER2 −	54 (68.4)

*AJCC* American Joint Committee of Cancer, *HER2* human epidermal growth factor receptor 2.

### Outcome

Median follow-up was 28.5 months (range, 4.2 to 108.5). Nine patients (9.3%) were lost to follow-up, 77 (79.4%) showed a progression or recurrent disease with a median delay of 10.3 months (range, 0.7 to 24.1) and 71 patients (73.2%) died during the follow-up period secondary to their GEJC with a median delay of 21.4 months (range, 2.6 to 84.1).

### Correlation analysis

**Figure 1:**
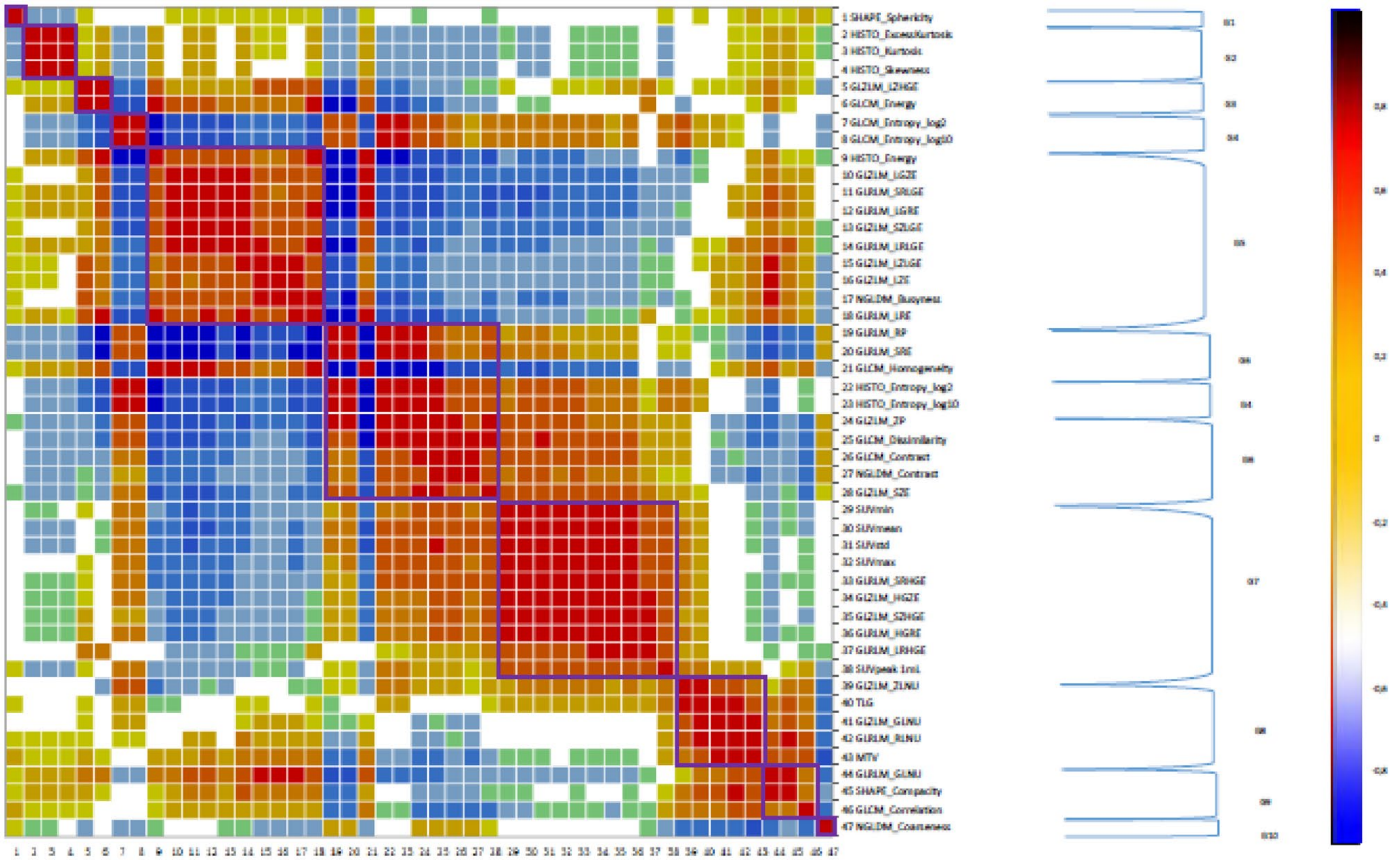
Pearson correlation heatmap for all textural features and Positron-Emission Tomography (PET) standard parameters.

Correlation analysis of 47 first-order radiomic indices and TFs (Supplemental Data 1) allowed the extraction of 10 groups with highly correlated parameters (r ≥ 0.8) (Fig. 1).

AJCC staging (I-II vs. III-IV) and treatment (surgery vs. no surgery) were significantly correlated (p = 0.022).

### Receiver operating characteristic (ROC) analysis

**Figure 2:**
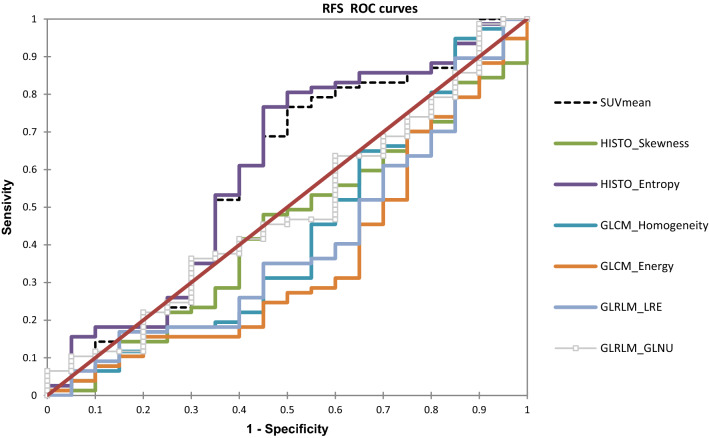
ROC curves with AUC to predict relapse free survival (RFS).

Three first-order radiomic indices (SUV_mean_, Histogram-Entropy and Histogram-Skewness) and 4 TFs (GLCM-Energy, GLCM-Homogeneity, GLRLM-LRE, GLRLM-GLNU) were significantly associated with RFS; 2 with a positive correlation: SUV_mean_ and Histogram-Entropy; 5 with a negative correlation: Histogram-Skewness, GLCM-Homogeneity, GLCM-Energy, GLRLM-LRE and GLRLM-GLNU (Fig. 2). No parameters from groups 1, 8 (including TLG and MTV) and 10 were significantly associated with survival in ROC analysis.

AUC, sensitivity, specificity and accuracy associated with different cutoff values are shown in Table 2.

**Table 2:** Predictive value of RFS according to ROC analysis.

Parameters	AUC	95% CI	Cutoff value	Se (%)	Sp (%)	Acc (%)
SUV_mean_	0.640	0.503–0.777	> 5.624	81.7	57.7	75.3
HISTO_skewness	0.614	0.489–0.739	< 1.428	90.1	30.8	74.2
HISTO_ entropy	0.621	0.484–0.759	> 0.897	84.5	53.8	76.3
GLCM_homogeneity	0.655	0.527–0.784	< 0.389	74.6	61.5	71.1
GLCM_energy	0.628	0.490–0.767	< 0.023	81.7	53.8	74.2
GLRLM_LRE	0.680	0.558–0.802	< 1.324	78.9	53.8	72.2
GLRLM_GLNU	0.629	0.507–0.750	< 23.915	52.1	76.9	58.8

*SUV* standardized uptake value, *HISTO* histogram, *GLCM* grey level co-occurrence matrix, *GLRLM* grey level run-length matrix, *LRE* long run emphasis, *GLNU* gray level non-uniformity for run, *AUC* area under curve, *CI* confidence interval, *Se* sensitivity, Sp specificity, *Acc* accuracy.

### Univariate analysis

#### Relapse-free survival

Table 3 demonstrates factors associated with RFS in univariate analysis.

**Table 3:** Univariate analysis of both clinical and textural factors with RFS.

Parameters	HR (95% CI)	*P*-value
Age (year)		
> 65y vs ≤ 65y	0.914 (0.623–1.527)	0.914
Sex		
Male vs. female	1.144 (0.604–2.168)	0.678
Histopathology		
Adenocarcinoma vs. SCC	1.107 (0.609–2.014)	0.739
AJCC stage		
III-IV vs. I-II	1.815 (1.045–3.153)	0.032*
Radio-chemotherapy		
Yes vs. no	1.349 (0.803–2.267)	0.256
Treatment		
Surgery vs. no surgery	0.379 (0.239–0.601)	< 0.0001*
SUVmean		
≥ 5.808 vs. < 5.808	1.866 (1.099–3.169)	0.019*
HISTO_Skewness		
≤ 1.468 vs. > 1.468	0.863 (0.455–1.636)	0.652
HISTO_ Entropy		
≥ 0.899 vs. < 0.899	1.983 (1.126–3.490)	0.015*
GLCM_Homogeneity		
≤ 0.389 vs. > 0.389	0.599 (0.369–0.971)	0.035*
GLCM_Energy		
≤ 0.023 vs. > 0.023	1.870 (1.089–3.208)	0.021*
GLRLM_LRE		
≤ 1.324 vs. > 1.324	1.489 (0.895–2.480)	0.123
GLRLM_GLNU		
≥ 23.915 vs. < 23.915	0.814 (0.520–1.276)	0.368

*HR* hazard ratio, *CI* confidence interval, *AJCC* American joint committee of cancer, *SCC* squamous cell carcinoma, *SUV* standardized uptake value, *HISTO* histogram, *GLCM* grey level co-occurrence matrix, *GLRLM* grey level run-length matrix, *LRE* long run emphasis, *GLNU* gray level non-uniformity for run.

*Significant *P*-value (< 0.05).

Patients who benefited from surgery had a significantly higher 2-year RFS rate (32.5% vs. 8.3%, p < 0.0001) whereas those with stage III-IV disease had a significantly lower rate (15.1% vs. 33.3%, p = 0.032).

Patients also had a significantly lower 2-year RFS rate in the SUV_mean_ ≥ 5.808 (15.7% vs. 33.3%, p = 0.019), Histogram-Entropy ≥ 0.899 (15.0% vs. 21.9%, p = 0,015) and GLCM-Energy ≤ 0.023 (34.6% vs. 12.7%, p = 0.021) subgroups while a significantly higher 2-year RFS rate was found in the GLCM-Homogeneity ≤ 0.389 (16.1% vs. 11.1%, p = 0,035) subgroup.

**Figure 3:**
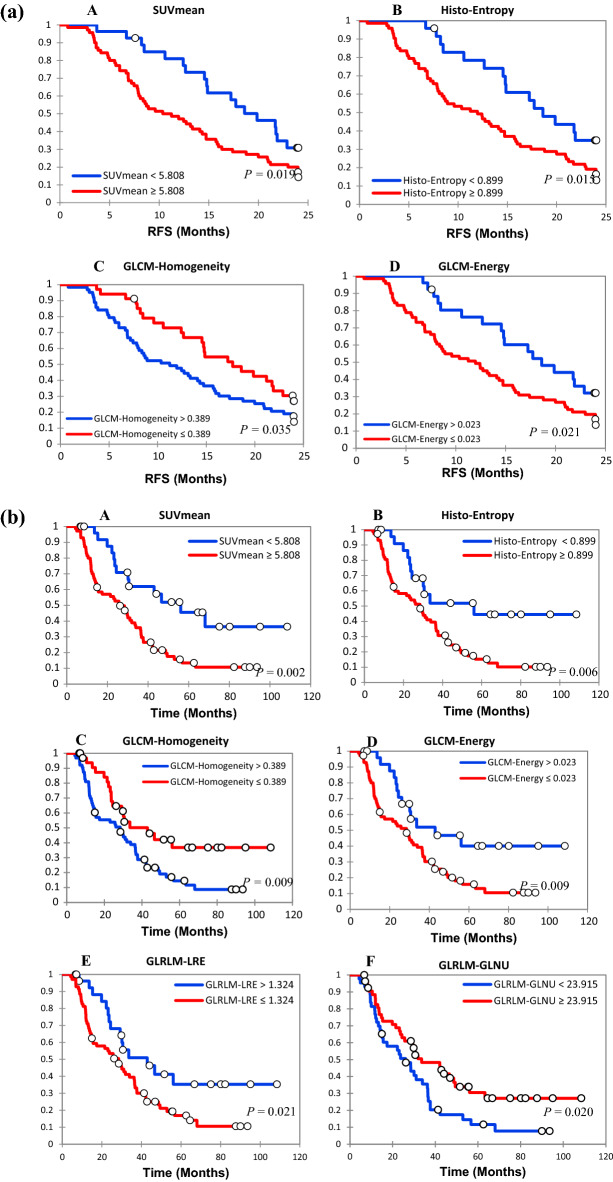
(**a**) Kaplan–Meier estimates of relapse free survival (RFS) between 2 groups according to optimal cut-off: A (SUVmean), B (Histo-Entropy), C (GLCM-Homogeneity), D (GLCM-Energy). (**b**) Kaplan–Meier estimates of overall survival (OS) between 2 groups according to optimal cut-off: A (SUVmean), B (Histo-Entropy), C (GLCM-Homogeneity), D (GLCM-Energy), E (GLRLM-LNE), F (GLRLM-GLNU).

Corresponding Kaplan–Meier curve are shown in Fig. 3a.

#### Overall survival

Table 4 demonstrates factors associated with OS in univariate analysis.

**Table 4:** Univariate analysis of both clinical and textural factors with OS.

Parameters	HR (95% CI)	*P*-value
Age (year)		
> 65y vs. ≤ 65y	1.095 (0.682–1.757)	0.707
Sex		
Male vs. female	2.061 (0.891–4.767)	0.084
Histopathology		
Adenocarcinoma vs. SCC	0.946 (0.495–1.805)	0.865
AJCC stage		
III-IV vs. I-II	1.793 (0.982–3.274)	0.054
Radio-chemotherapy		
Yes vs. no	0.923 (0.545–1.561)	0.764
Treatment		
Surgery vs. no surgery	0.406 (0.251–0.654)	0.000*
SUVmean		
≥ 5.808 vs. < 5.808	2.558 (1.394–4.693)	0.002*
HISTO_skewness		
≤ 1.468 vs. > 1.468	0.833 (0.381–1.822)	0.646
HISTO_ entropy		
≥ 0.899 vs. < 0.899	2.397 (1.257–4.573)	0.006*
GLCM_homogeneity		
≤ 0.389 vs. > 0.389	0.493 (0.288–0.844)	0.009*
GLCM_energy		
≤ 0.023 vs. > 0.023	2.194 (1.199–4.012)	0.009*
GLRLM_LRE		
≤ 1.324 vs > 1.324	1.936 (1.092- 3.431)	0.021*
GLRLM_GLNU		
≥ 23.915 vs. < 23.915	0.576 (0.360–0.921)	0.020*

*HR* hazard ratio, *CI* confidence interval, *AJCC* American joint committee of cancer, *SCC* squamous cell carcinoma, *SUV* standardized uptake value, *HISTO* histogram, *GLCM* grey level co-occurrence matrix, *GLRLM* grey level run-length matrix, *LRE* long run emphasis, *GLNU* gray level non-uniformity for run.

*Significant *P*-value (< 0.05).

A significantly higher 5-year OS rate was seen in patients who underwent surgery surgery (36.7% vs. 16.6%, p < 0.001) and with GLCM-Homogeneity ≤ 0.389 (47.1% vs. 15.9%, p = 0.009) and GLRLM-GLNU ≥ 23.915 (37.0% vs. 15.9%, p = 0.020).

A significantly lower 5-year OS rate was seen if they had a SUV_mean_ ≥ 5.808 (17.1% vs. 51.8%, p = 0.002), a Histogram-Entropy ≥ 0.899 (17.8% vs. 54.2%, p = 0.006), a GLCM-Energy ≤ 0.023 (18.3% vs. 50%, p = 0.009) and a GLRLM-LRE ≤ 1.324 (18.8% vs. 46.4%, p = 0.021).

Corresponding Kaplan–Meier curves are shown in Fig. 3b.

#### Adenocarcinoma subgroup and HER2 expression

HER2 positive patients had a non-significantly lower 2-year RFS rate than HER2 negative ones (p = 0.32).

### Multivariate analysis

All prognostic factors with p–value < 0.20 in the univariate analysis were included in the multivariate model to evaluate their interaction and joint effect. Because of the high correlation between AJCC staging and surgery, only surgery was integrated in the model and was arbitrarily chosen over AJCC staging because of its higher predictive value in univariate analysis.

For RFS, Cox proportional hazard regression model found that surgery (HR = 0.376; 95% CI 0.237–0.597; p = 0.000) and Histogram-Entropy (HR = 2.004; 95% CI 1.137–3.535; p = 0.016) were independent predictive factors (Table 5).

**Table 5:** Multivariate Cox regression analysis of RFS.

Parameters	HR (95% CI)	*P*-value
AJCC stage		
III-IV vs. I-II	1.374 (0.782–2.416)	0.270
Treatment		
surgery vs. no surgery	0.376 (0.237–0.597)	0.000*
SUVmean		
≥ 5.808 vs. < 5.808	0.946 (0.371–2.415)	0.908
HISTO_ Entropy		
≥ 0.899 vs. < 0.899	2.004 (1.137–3.535)	0.016*
GLCM_Homogeneity		
≤ 0.389 vs. > 0.389	0.827 (0.404–1.693)	0.604
GLCM_Energy		
≤ 0.023 vs. > 0.023	1.142 (0.226–5.783)	0.872

*HR* hazard ratio, *CI* confidence interval, *AJCC* American joint committee of cancer, *SUV* standardized uptake value, *HISTO* histogram, *GLCM* grey level co-occurrence matrix.

*Significant *P*-value (< 0.05).

For OS, Cox proportional hazard regression model found that surgery (HR = 0.375; 95% CI 0.227–0.620; p < 0.001), SUV_mean_ ≥ 5.808 (HR = 2.201; 95% CI 1.120–4.326; p = 0.022) and Histogram-Entropy ≥ 0.899 (HR = 2.397; 95% CI 1.257–4.573; p = 0.006) were independent predictive factors (Table 6).

**Table 6:** Multivariate Cox regression analysis of OS.

Parameters	HR (95% CI)	*P*-value
Sex		
Male vs. female	2.125 (0.905–4.998)	0.083
AJCC stage		
III-IV vs. I-II	1.045 (0.532–2.052)	0.899
Treatment		
Surgery vs. no surgery	0.375 (0.227–0.620)	0.000*
SUVmean		
≥ 5.808 vs. < 5.808	2.201 (1.120–4.326)	0.022*
HISTO_ entropy		
≥ 0.899 vs. < 0.899	2.397 (1.257–4.573)	0.006*
GLCM_homogeneity		
≤ 0.389 vs. > 0.389	0.616 (0.180–2.102)	0.439
GLCM_energy		
≤ 0.023 vs. > 0.023	0.696 (0.130–3.721)	0.672
GLRLM_LRE		
≤ 1.324 vs > 1.324	0.790 (0.320–1.951)	0.610
GLRLM_GLNU		
≥ 23.915 vs. < 23.915	0.641 (0.376–1.094)	0.103

*HR* hazard ratio, *CI* confidence interval, *AJCC* American joint committee of cancer, *SCC* squamous cell carcinoma, *SUV* standardized uptake value, *HISTO* histogram, *GLCM* grey level co-occurrence matrix, *GLRLM* grey level run-length matrix, *LRE* long run emphasis, *GLNU* gray level non-uniformity for run.

*Significant *P*-value (< 0.05).

The results of multivariate analysis were confirmed in the internal validation cohort for the primary endpoint only (Supplemental Data 2).

## Discussion

To our knowledge, this study is the first to clinically assess the prognostic value of both first-order PET indices and TFs in a wide cohort of patients with GEJC. Only one review has specifically investigated the prognostic value of PET parameters in selected GEJC, but only focused on first-order PET indices^14^. Predicting prognosis before treatment initiation remains crucial to optimize the management patients with GEJC^9,10^.

Regarding the first-order PET indices, SUV_max_ and SUV_mean_ were the most widely parameters investigated in patients with esophageal^6^ and gastric^7^ cancers. However, no SUV threshold had been clearly demonstrated to dichotomize patients in different subgroups of prognosis within meta-analysis. Thus, the trend emerging from literature is that SUV_max_ is a predictive marker of survival (RFS and OS) but with a robustness that varies and baseline SUV_max_ appears as a prognostic biomarker but in selected groups of patients with stage I-II or III disease, therefore eligible for surgery or CRT. Large prospective studies, especially including stage IV disease, would be important to confirm these results because 20 to 30% of patients with esophageal cancer, including GEJC, have distant metastasis at diagnosis^2^. In our study, the SUV_mean_ parameter was correlated with RFS in univariate analysis and remained an independent prognostic factor of OS with a cut-off of 5.808. It was chosen because it was more discriminating (p = 0.045) than both SUV_max_ (p = 0.125) and SUV_peak_ (p = 0.141) (G7, Fig. 1) to predict the outcome. SUV_mean_ is defined as the mean intensity in a lesion and is a numerical reading of the tracer uptake based on the injected dose and the patient’s body weight. It reflects the metabolic activity of most of the tumor. SUVmean provides informations about intrinsic lesion characteristics, related to tumor grading, biological factors, and the presence of hypoxic or necrotic areas; in addition, it is considered more robust to potential noise bias associated with SUVmax but has the disadvantage of being highly dependent on VOI size^15–17^. There is no literature specifically assessing the prognostic value of SUV_mean_ in patients with GEJC but only several studies in esophageal or gastric cancers. In a population of 21 patients with both esophageal SCC and ADC histologies of different stages, Ganeshan et al. showed that there was a significant correlation between survival and SUV_mean_ (cut-off of 3.524) in univariate analysis (p = 0.0032)^18^. Nevertheless, in other series of 50 patients with esophageal SCC and ADC who underwent an exclusive concomitant radio-chemotherapy, a pre-therapeutic SUV_mean_ (cut-off value of 5) was not significant for survival (p = 0.06)^19^.

In our analysis of textural features, we showed that Histogram-Entropy was an independent prognostic factor of RFS (p = 0.016) and OS (p = 0.006) with a cut-off of 0.899. To date, 10 studies were performed in patients with esophagus and gastric cancers to investigate the prognostic value of second and higher-order features of the radiomic approach^10,20^. However, these studies were heterogeneous in terms of design (i.e. predictive value of the pre-therapeutic TFs or evaluation of pre-post therapeutic value of TFs), treatment (mainly CRT) and endpoints (response rate for most of these studies). In a retrospective study of 44 patients with esophageal SCC treated with neoadjuvant chemoradiotherapy and surgery, Chen et al. also found that Histogram-Entropy was significant to predict response to CRT based on pretreatment PET (p = 0.009)^20^. In a retrospective study of 41 patients with esophagus cancer, Tixier et al. also found that GLCM-Entropy was significant to predict response to CRT based on pretreatment PET (Se = 79%, Sp = 82%)^21^. This TF was highly correlated to GLCM-Entropy in our subgroup G4 (Fig. 1). However, this previous study did not investigate its prognostic value for RFS and/or OS. In a cohort study of 403 patients with esophageal cancer including 110 GEJC, Foley et al. compared the prognostic value of 8 first-order PET indices and 8 TFs with the current staging methods (age, stage and treatment) for OS prediction. They found, in addition to TLG (p = 0.013), that Histogram-Energy (p = 0.011) and Histogram-Kurtosis (p = 0.017) were independent prognostic factors of survival^11^. Moreover, Nakajo et al. reported in their study of 52 patients with advanced SCC esophagus cancer, that both GLZLM-GLNU and GLZLM-ZLNU were prognosis factors for survival in univariate analysis (p = 0.013 and p = 0.013, respectively) with a high correlation to volumetric PET parameters (MTV, TLG)^22^. Nevertheless, GLCM-Entropy was not associated with OS in this study (p = 0.18). Then, in a study of 36 patients with esophagus cancer including both SCC and adenocarcinoma histologies, Zhang et al. found that GLCM-Energy was not a prognostic^23^. Finally, in a study of 65 patients with esophagus cancer including both SCC and adenocarcinoma histologies, Desbordes et al. found that GLCM-Homogeneity was a predictor of response to treatment^24^. In our study, GLCM-Homogeneity (p = 0.035) and GCLM-Energy (p = 0.021) were correlated with RFS only in univariate analysis. Thus, these findings appear to be mainly consistent with the literature. The few discrepancy results might be explained by the selected population of GEJC we studied.

Assessing the evolution of TFs between pre- and post-therapeutic FDG-PET/CT may also be an interesting way of research^10^. Indeed, van Rossum et al. showed that change in GLCM-Entropy was better to predict response compared to the clinical model alone in 217 patients with esophageal adenocarcinomas undergoing preoperative CRT^9^.

As above mentioned, one of the challenges is to optimize patients care with personalized treatment. For this purpose, to design combination of clinicopathological parameters with imaging radiomic features seems interesting. This approach begins to develop and has already been evaluated, especially in breast cancers. In this way, Aoji et al.^25^ found that pre-therapeutic SUV_max_ > 6.0 was associated with a poor RFS and OS in 262 luminal-type (hormonal receptor-positive) breast cancer patients. These findings are important because luminal-type breast cancer is generally managed using only hormonal therapy and identifying patients with poor prognosis in such population would enable the use of a more intensive treatment right from the beginning. In another illustration of treatment personalization strategy, Humbert et al. showed that ΔSUVmax of the primary tumor after only one cycle of docetaxel plus trastuzumab regimen was able to predict response in patients with HER2 + locally advanced breast cancer, with possibility to improve response by adding pertuzumab^26^.

In our study, we have performed a subgroup analysis in patients with adenocarcinoma according to their HER2 status, overexpressed from 7 to 34% in GEJC^2^. HER2 + and HER2- patients did not show a statistically significant difference in RFS and OS, probably due to stage and tumor heterogeneities.

Methodological aspect in radiomic studies remains essential to ensure the reproducibility of results for clinical practice^8^. In our method, we used an absolute intensity resampling of 0–30 of the SUV with a fixed number of 64 bins. These grey levels and absolute intensity resampling were based on the results of previous studies assessing TFs robustness in FDG PET/CT^27,28^. Otherwise, SUV 0–30 corresponds to the typical range of tumor SUVs encountered in GEJC^6,7^. Thus, we chose a reproducible relative SUV threshold (40%SUVmax) as tumor delineation method. Indeed, Guezennec et al. reported a high interobserver reproducibility with this method to calculate TFs in an analysis of 43 head and neck cancers, revealing an excellent agreement for 3 indices (GLCM-Homogeneity, GLCM-Correlation and GLCM-Entropy) with an intraclass correlation coefficient higher than 0.90^29^. Nevertheless, they found a high variability of contouring in comparison with the gradient-based method. The FLAB (fuzzy locally adaptive Bayesian) automatic method is an interesting model that showed an improvement of true tumor volume estimation in esophageal lesions^9,30^. Moreover, Hatt et al. highlighted that FLAB isocontouring did not significantly affected the robustness of GLCM-Entropy, that was correlated with both RFS and OS in our univariate analysis^30^. This confirms our findings about Histogram-Entropy that was highly correlated to GLCM-Entropy, appearing to be a robust TF to assess prognostic value of esophageal cancers. However, to avoid the lack of reproducibility and to allow harmonization of practice, recent standardization of radiomic procedures has been proposed^31,32^. The future of radiomics will undeniably involve artificial intelligence, which will further strengthen its robustness^33^.

Our study presents several limitations. Firstly, our cohort is heterogeneous with high proportion of advanced stages, different histological types including HER2 status and non-uniformity in treatment regimens, but reflects routine practice. Secondly, our study was retrospective and conducted in a single center with a risk of selection bias. However, studying PET radiomic in multicentric trials remains difficult in terms of methodology, even if harmonization procedures between the different machines are available to limit the variability resulting from different image resolutions and reconstructions^32,34^. Thirdly, there is near 10% of patients lost to follow-up and follow-up is only 3 years; the disease has a poor prognosis so there are few survivors beyond. Finally, our findings were tested only in an internal validation cohort and were confirmed for RFS unlike OS. Despite these limitations, we obtained highly significant results demonstrating a prognostic role of FDG-PET/CT radiomic approach combining first-order statistics and TFs in patients with GEJC, opening perspectives of combining scoring systems^35^.

## Conclusion

Histogram-Entropy and SUV_mean_ were independently associated with survival in patients with GEJC. Thus, TFs showed a real potential to be a clinically useful imaging biomarker providing additional prognostic information for patients’ selection. The complementary role of first-order PET parameters (i.e. SUV-based and volumetric parameters) and TFs predicting patients outcome appear complex and warranted prospective studies.

## Supplementary Information

Supplementary Tables.

## Data Availability

The datasets analysed during the current study are available from the corresponding author on reasonable request.
