# Author Correction: Prognostic value of pre-therapeutic FDG-PET radiomic analysis in gastro-esophageal junction cancer

**DOI:** 10.1038/s41598-023-33877-7

**Published:** 2023-04-24

**Authors:** Karim Amrane, Philippe Thuillier, David Bourhis, Coline Le Meur, Chloe Quere, Jean-Christophe Leclere, Marc Ferec, Veronique Jestin-Le Tallec, Laurent Doucet, Pierre Alemany, Pierre-Yves Salaun, Jean-Philippe Metges, Ulrike Schick, Ronan Abgral

**Affiliations:** 1 Department of Oncology, Regional Hospital of Morlaix, Morlaix, France; 2 Department of Endocrinology, University Hospital of Brest, Brest, France; 3 UMR Inserm 1304 GETBO, IFR 148, University of Western Brittany, Brest, France; 4 Department of Nuclear Medicine, University Hospital of Brest, 2 Avenue Foch, 29609 Brest Cedex, France; 5 Department of Oncology, University Hospital of Brest, Brest, France; 6 Department of Otorhinolaryngology, University Hospital of Brest, Brest, France; 7 Department of Gastroenterology, Regional Hospital of Morlaix, Morlaix, France; 8 Department of Oncology, Pasteur Clinic, Brest, France; 9 Department of Pathology, University Hospital of Brest, Brest, France; 10 Department of Pathology, Ouestpathology Brest, Brest, France; 11 Department of Radiotherapy, University Hospital of Brest, Brest, France

Correction to: *Scientific Reports* https://doi.org/10.1038/s41598-023-31587-8, published online 08 April 2023

Reference 12 in the original version of this Article was omitted. The reference is listed below.

12. Nioche C., Orlhac F., Boughdad S., et al. LIFEx: a freeware for radiomic feature calculation in multimodality imaging to accelerate advances in the characterization of tumor heterogeneity*. Cancer Research* **78**(16):4786–4789 (2018).

In the Materials and methods section, under the subheading ‘Images analysis’,

“All primary tumors were analyzed using LIFEx software (http://www.lifexsoft.org) on PET imaging.

”now reads:

“All primary tumors were analyzed using LIFEx software (http://www.lifexsoft.org)^12^ on PET imaging.”

The References have been renumbered.

The original Article has been corrected.

