# Supplementary material for: Prognostic value of pre-therapeutic FDG-PET radiomic analysis in gastro-esophageal junction cancer
Source: Sci Rep. 2023 Apr 8;13:5789. doi: 10.1038/s41598-023-31587-8 (PMC10082755; doi:10.1038/s41598-023-31587-8)
Supplement: Supplementary file 1 — Supplementary Tables. [file 41598_2023_31587_MOESM1_ESM.docx]

**SUPPLEMENTAL DATA 1 (internal validation cohort)**

**Table S0:** Groups of highly correlated indices**.**

| **Groups** | **Radiomic features** | **ACC**  **(mean ± SD)** |
| --- | --- | --- |
| 1 | SHAPE_Sphericity | - |
| 2 | HISTO_Skewness, HISTO_Kurtosis, HISTO_ExcessKurtosis | 0.956 ± 0.033 |
| 3 | GLZLM_LZHGE, GLCM_Energy | 0.905 ± 0.109 |
| 4 | GLCM_Entropy_log2, GLCM_Entropy_log10, HISTO_Entropy_log2, HISTO_Entropy_log10 | 0.950 ± 0.050 |
| 5 | HISTO_Energy, GLRLM_LRE, GLRLM_SRLGE, GLRLM_LGRE, GLRLM_LRLGE, GLZLM_LZE, GLZLM_LGZE, GLZLM_SZLGE, GLZLM_LZLGE, NGLDM_Busyness | 0.800 ± 0.123 |
| 6 | GLCM_Homogeneity, GLCM_Dissimilarity, GLCM_Contrast, NGLDM_Contrast, GLRLM_SRE, GLRLM_RP, GLZLM_SZE, GLZLM_ZP | 0.816 ± 0.145 |
| 7 | SUVmin, SUVmean, SUVstd, SUVmax, SUVpeak, GLRLM_HGRE, GLRLM_SRHGE, GLRLM_LRHGE, GLZLM_HGZE, GLZLM_SZHGE | 0.924 ± 0.078 |
| 8 | TLG (g), MTV (mL), GLZLM_GLNU, GLZLM_ZLNU, GLRLM_RLNU | 0.811 ± 0.152 |
| 9 | SHAPE_Compacity, GLCM_Correlation, GLRLM_GLNU | 0.792 ± 0.191 |
| 10 | NGLDM_Coarseness | - |

*(Abbreviations: GLCM= grey level co-occurrence matrices; GLRLM= grey-level run length matrix; GLZLM= grey-level zone length matrix; NGLDM= neighborhood grey-level different matrix; SUV: standardized uptake value; MTV= metabolic tumor volume; TLG= total lesion glycolysis; ACC=absolute correlation coefficient).*

**SUPPLEMENTAL DATA 2**

| **Characteristics** | **No of patients (n=33)** |
| --- | --- |
| **Age years, median (range)** | 65 (43-87) |
| **Sex (male / female)** | 28/5 |
| **Histology, n (%)** |  |
| Squamous cell | 4 (12.1) |
| Adenocarcinoma | 29 (87.9) |
| **AJCC stage, n (%)** |  |
| I (T1a N0 M0) / (T1b N0 M0) | 33 (9.1) |
| II (T2 N0 M0) / (T1 N1 M0) / (T3 N0 M0) | 05 (15.1) |
| III (T1 N2 M0) / (T2 N1 M0) / (T2 N2 M0) / (T3 N1/N2 M0) /  (T4a N0/N1 M0) | 10 (30.3) |
| IV (T4a N2 M0) / (T4b Nx M0) / (Tx N3 M0) / (Tx, Nx, M1) | 15 (45.5) |
| **Treatment, n (%)** |  |
| Surgery alone | 03 (9.1) |
| Neo and adjuvant treatment +surgery | 10 (30.3) |
| Radiochemotherapy (RCT) | 09 (27.3) |
| Chemotherapy alone | 10 (30.3) |
| Best supportive care | 01 (3.0) |
|  | Adenocarcinoma (n=29) |
| **Siewert classification (adenocarcinoma type), n (%)** |  |
| I | 12 (41.4) |
| II | 10 (34.5) |
| III | 07 (24.1) |
| **Mutations (for adenocarcinoma), n (%)** |  |
| HER2 + | 012 (41.4) |
| HER2 - | 17 (58.6) |

**Table S1:** Characteristics of patients at baseline.

**Table S2a:** Analysis of both clinical and textural factors with RFS.

| **Parameters** | HR (95% CI) | *P*-value |
| --- | --- | --- |
| **Treatment**  surgery vs no surgery | 0.324 (0.136-0.772) | 0.008* |
| **HISTO_** **Entropy**  ≥ 0.899vs < 0.899 | 3.543 (1.047-11.585) | 0.030* |

*(Abbreviations: HR = Hazard Ratio; CI = Confidence Interval; HISTO = Histogram;)*.

*Significant *P*-value (< 0.05).

**Table S2b:** Analysis of both clinical and textural factors with OS.

| **Parameters** | HR (95% CI) | *P*-value |
| --- | --- | --- |
| **Treatment**  surgery vs no surgery | 0.642 (0.417-0.986) | 0.048* |
| **SUVmean**  ≥ 5.808 vs < 5.808 | 2.506 (1.029-6.767) | 0.049* |
| **HISTO_** **Entropy**  ≥ 0.899vs < 0.899 | 3.270 (1.448-11.092) | 0.045* |

*(Abbreviations: HR = Hazard Ratio; CI = Confidence Interval; SUV = Standardized Uptake Value; HISTO = Histogram)*.

*Significant *P*-value (< 0.05).
